# Effects of traditional Chinese culture-based bibliotherapy on the spiritual health of patients with liver cancer

**DOI:** 10.1007/s00520-023-08154-y

**Published:** 2023-11-10

**Authors:** Ling Huang, Honghui Zhang, Yuting Xiao, Qian Li, Xiu Huang, Lihui Li, Shan Xiao, Ou Li, Le Wang

**Affiliations:** 1 Department of Nursing, Hunan Provincial People’s Hospital, The First-Affiliated Hospital of Hunan Normal University, Changsha, Hunan China; 2 Department of Hepatobiliary Surgery, Hunan Provincial People’s Hospital, The First-Affiliated Hospital of Hunan Normal University, Changsha, Hunan China

**Keywords:** Spiritual health, Bibliotherapy, Liver cancer, Chinese traditional culture

## Abstract

**Background:**

Liver cancer is a serious global health problem and is associated with poor spiritual health. Bibliotherapy is beneficial in improving health outcomes in cancer patients, yet there is a lack of empirical evidence of its effect on the spiritual health of liver cancer patients in China. The study aimed to investigate the effects of bibliotherapy based on Chinese traditional culture on the spiritual health of patients with liver cancer in China. This study was approved by the Ethics Committee of Hunan Normal University School of Medicine and registered with the Chinese Clinical Trial Registry with the registration (No: 2021260), which registration in June 30th 2021.

**Methods:**

A total of 60 patients with liver cancer were divided into the intervention group (*n* = 30) and the control group (*n* = 30) through WeChat. The intervention group received bibliotherapy therapy based on traditional Chinese culture, while the control group received routine care. Spiritual health was assessed using the Spiritual Attitude and Involvement List (SAIL) and compared before and after the intervention between the two groups. The chi-square test and *t*-test were used to analyze the intervention effects.

**Results:**

The two groups were comparable in all baseline characteristics including the SAIL score. After 5 weeks of intervention, the score of SAIL increased significantly from 96.76 ± 15.08 to 106.93 ± 13.82 in the intervention group (*t* =  − 29.380, *p* < 0.001), while no significant difference in SAIL score was observed in the control group (from 95.27 ± 16.40 to 95.31 ± 16.24, *t* =  − 0.189, *p* = 0.852). Similar patterns were also observed in its three dimensions of connecting with oneself, connecting with the environment, and connecting with transcendence.

**Conclusions:**

Our study showed that bibliotherapy based on traditional Chinese culture using the WeChat platform can greatly improve the spiritual health of patients with liver cancer and has the potential to be widely applied to cancer patients to improve their well-being.

## Introduction

Liver cancer is a serious global health concern, ranking as the sixth most common cancer and the third leading cause of cancer-related death worldwide in 2020 [1]. In China, liver cancer was the fourth most common cancer, with an estimated 388,800 cases and over 70% were males, according to the most recent data from the National Cancer Center [2]. With a large population base, China accounted for about half of the global burden of liver cancer [2]. Liver cancer has the second-lowest survival rate among all cancers in China, with a 5-year survival rate of 12.1% [3]. The high prevalence rate and low survival rate of liver cancer pose significant challenges to China’s cancer prevention and control.

Liver cancer negatively affects various aspects of the patient’s well-being, among which spiritual health is an important yet less studied area. Spiritual health is a multidimensional concept that covers pain and death, guilt and shame, reconciliation and forgiveness, freedom and responsibility, hope and despair, love, and joy [4]. The World Health Organization emphasizes that spiritual health is another important aspect in addition to mental health, physical health, and social health, and is one of the four pillars of whole-person health [5]. Spiritual health is especially important when faced with a life-threatening disease such as cancer, with studies showing that more than 90% of cancer patients had at least one spiritual health need, including seeking inner peace, being full of hope, finding meaning in life, giving or receiving love, being recognized, and being forgiven [6]. Patients with liver cancer are at lower levels of spiritual health due to certain characteristics of the disease, such as late diagnosis, infection with hepatitis B, difficult treatment, repeated disease course, and long treatment cycle, which may cause stigma, stress, despair, depression, and anxiety among the patients. Lower spiritual health is associated with lower quality of life and poorer physical and mental health [7], while improvement of spiritual health can significantly improve the immune function of patients with liver cancer, reduce oxidative stress, and improve the quality of life [8]. It is thus of paramount importance to improve the spiritual health of liver cancer patients.

Bibliotherapy is an intervention that “uses literary texts and guided group discussion to promote self-understanding, cognitive, emotional, behavioral, and spiritual change in patients” [9]. It is built on self-help books and applies either cognitive or behavioral therapy, with a special focus on the unconscious, emotional, and spiritual aspects of human beings [10]. For over a century, bibliotherapy has been widely applied in various therapies among various populations with various conditions to support healing and well-being [11]. Bibliotherapy is especially beneficial for patients with cancer, with a recent literature review showing its efficacy in improving a wide array of patient outcomes, including better coping, more social support, higher self-efficacy and self-esteem, better interpersonal relationships, reduced level of psychological distress, and improved quality of life [12].

However, evidence on the effects of bibliotherapy on the spiritual health of liver cancer patients in China is limited. In addition, most previous studies on bibliotherapy were conducted in a Western cultural context. As spiritual health varies by religion and culture, the Western understanding of bibliotherapy and spiritual health may not apply to an Asian country such as China. For instance, research on spiritual health in the USA has mostly revolved around the dominant Christian faith culture [13], which may be different from Muslim, or other cultural backgrounds and religions [14, 15]. It is thus necessary to explore spiritual health from different cultural perspectives, especially the less-studied Asian cultures [16]. In China, there are many health education measures that are implemented through WeChat and are also used for spiritual health improvement [17]. And in traditional Chinese culture, there are also many connotations of spiritual health [18]. In light of the above-mentioned limitations, we conducted the current study to explore the effect of bibliotherapy based on traditional Chinese culture on the spiritual health of liver cancer patients in China.

## Methods

### Study design, participants, and procedure

This was a quasi-experimental study conducted in a tertiary hospital in Changsha City, Hunan Province, from August to October 2021. All patients between the ages of 16 and 65 years who received treatment for liver cancer at the Department of Hepatic Surgery of the Hospital were included in the study. The inclusion criteria included as follows: (1) meeting the diagnostic criteria for liver cancer and knowing their own conditions; (2) being aged between 16 and 65 years old; (3) signing the informed consent; (4) being able to surf the Internet and use smartphones; (5) with clear consciousness and able to communicate in Chinese. The exclusion criteria included as follows: (1) with severe complications and cannot take care of themselves; (2) with mental problems or unclear consciousness and cannot cooperate with the researcher.

A total of 60 patients with liver cancer who were recently discharged from the hospital were recruited in the study. The total sample was divided into the intervention group and the control group. The intervention group received bibliotherapy therapy based on traditional Chinese culture through the WeChat platform, while the control group received routine care. We compared spiritual health between the experimental group and the control group before and after the intervention and explored the effects of bibliotherapy therapy on the spiritual health of patients with liver cancer.

This study was approved by the Ethics Committee of Hunan Normal University School of Medicine and registered with the Chinese Clinical Trial Registry with the registration (No: 2021260), which registration in June 30th 2021. The research subjects read the informed consent form before participating in the research and were informed of the purpose, significance, and form of participation in the research. The patient’s decision to participate in the study was voluntary, and informed consent was obtained for treatment.

### Interventions

Both the control group and the experimental group received a 5-week intervention program through WeChat from October to November 2021.

1. Control group
  Thirty patients in the control group were assigned to the “health education” WeChat group. They received regular health education through WeChat, including diet management, exercise management, and complication management. Every week, 3–5 health-related education messages were regularly sent to the participants. A head nurse was in charge of the WeChat group, providing consultation on nursing-related issues, encouraging patients to express their problems, and answering patients’ questions in a timely manner.
2. Intervention group
  Thirty patients in the intervention group were assigned to the “bibliotherapy” WeChat group. Apart from the regular health education of the control group, the intervention group received bibliotherapy therapy based on traditional Chinese culture to help patients relive the knowledge of Confucianism, Taoism, and Traditional Chinese Medicine. Participants were invited to follow the “Spirituality and Health” WeChat Official Account (WOA) developed by the research team by scanning a QR code. The research team created and determined the contents of the WOA publications according to the patient’s needs, with Confucianism, Taoism, and Traditional Chinese Medicine as the major themes. The contents of the articles refer to the original texts of “The Analects of Confucius,” “Tao Te Ching,” and “The Yellow Emperor’s Classic of Internal Medicine.” The number of words in each article was between 500 and 700, and the reading time was within 10 min. The research team forwarded the WOA publications to the intervention group from 10:00 to 12:00 every morning and encouraged patients to post their comments after reading and to engage in discussion in the WeChat group. Participants completed reading and signed in to the group before 21:00 every day. Those who did not sign in were reminded by the research team through WeChat. Details about the intervention program by week were shown below:

- In the first week, the participants were given a basic introduction to spiritual health, including its concept, origin, development, significance, and ways to improve it.
- The second week was focused on Confucian culture. The participants were briefly introduced to the Confucian culture, which aroused their thinking about Confucian culture. Some contents related to spiritual health in works such as “The Analects of Confucius” and “Mencius” were quoted and analyzed in the WeChat group to guide thinking and discussion.
- The third week was focused on Taoist culture. The participants were briefly introduced to the cultural effect of Taoist thought on health, which aroused the participants’ attention to Taoist thought. Some ideas related to spiritual health in Tao Te Ching were cited to guide participants to establish a positive life attitude, with a focus on returning to nature. In addition, the unique qigong was introduced in Taoism and participants were encouraged to adjust their breathing to feel the peace of mind.
- The fourth week was themed on Traditional Chinese Medicine. The participants were briefly introduced to some Traditional Chinese Medicine knowledge to feel the wisdom of traditional Chinese culture. The contents of “Huangdi Neijing” were quoted to teach the participants to adjust the order of life according to the laws of nature. Participants were encouraged to establish a healthy lifestyle by eating and drinking regularly, regulating emotions, and cultivating spirits.
- The fifth week entered the end of the study, with a focus on summarizing the knowledge learned to help participants further master the connotation of traditional Chinese culture.

### Evaluation

Spiritual health was assessed by the Spiritual Attitude and Involvement List (SAIL). The original SAIL was developed by Dutch scholars de Jager Meezenbroek et al. [19] to measure spiritual well-being on seven subscales: (1) meaningfulness, (2) trust, (3) acceptance, (4) caring for others, (5) connectedness with nature, (6) transcendent experiences, and (7) spiritual activities. The seven subscales were further classified into three dimensions: connection with oneself (subscales 1, 2, and 3), connection with nature (subscales 4 and 5), and connection with transcendence (subscales 6 and 7). It includes 26 items rated on a 6-point Likert scale ranging from 1 (not at all) to 6 (to a very high degree). Respondents were asked to rate the degree to which each specific statement about spiritual well-being applies to themselves (e.g., “I know what my position is in life”). The total score ranges from 26 to 156, with a higher score indicating better spiritual health. In 2020, the scale was translated into Chinese by He Wenqi, with one subscale “transcendent experiences” deleted, while the three dimensions remained the same [20]. The Chinese version of SAIL has demonstrated good reliability and validity. In the current study, the SAIL showed good internal consistency with a Cronbach’s alpha coefficient of 0.85.

### Statistical analysis

All statistical analyses were performed using the SPSS version 22.0. Categorical variables were expressed as numbers and percentages and compared using the chi squared or Fisher’s exact test. Continuous variables were expressed as means and medians and compared using the two-sample independent or paired sample *t*-test. All the statistical tests were 2-sided and *p* < 0.05 was considered statistically significant.

## Results

### Study population

Table 1 shows the socio-demographic characteristics of the study population and their comparisons between the intervention group and the control group. The final sample included 56 liver cancer patients. Most of the patients were males (68%), aged between 41 and 50 years old (57%), married (77%), and non-religious (73%). Approximately half of the patients had a college education (50%) and were employed (52%). There was no significant difference in terms of the socio-demographic characteristics between the intervention group and control group, with all *p* > 0.05. indicating that the two groups of patients were comparable. In terms of awareness of Chinese traditional culture, 51% of patients had little understanding of traditional culture, 31% were relatively familiar with traditional culture, and 41% expressed interest in traditional culture.

**Table 1:** Baseline comparison of socio-demographic characteristics between the two groups

Variable	Intervention group (*n* = 30)	Control group (*n* = 26)	*χ*^2^	*p*
	Number of cases/percentages (%)	Number of cases/percentages (%)		
Gender			0.042	0.838
Male	20/66.7	18/69.2		
Female	10/33.3	8/30.8		
Age			38.578	0.164
< 30	4/13.3	4/15.4		
31–40	7/23.3	4/15.4		
41–50	12/40.0	10/38.5		
51–65	7/23.3	8/30.7		
Marital status			1.749	0.626
Married	24/80.0	19/73.1		
Unmarried	4/13.3	5/19.2		
Divorced	1/3.3	2/7.7		
Widowed	1/3.3	0		
Educational level			4	0.289
Middle school and below	1/3.3	5/19.2		
High school	11/36.7	6/23.1		
College	16/53.3	12/46.2		
Graduate and above	2/6.7	3/11.5		
Religious belief			1.949	0.583
None	21/70.0	20/76.9		
Buddhism	6/20.0	3/11.5		
Christianity	3/10.0	2/7.7		
Other	0	1/3.8		
Employment			10.252	0.680
Unemployed	7/23.3	0		
Employed	12/40.0	17/65.3		
Retired	5/16.7	2/7.7		
Student	6/20.0	7/26.9		

### Baseline comparison of spiritual health between the two groups before intervention

Before the formal intervention, the SAIL was administered to both groups of patients to assess their spiritual health. The scores of the SAIL and its three dimensions were normally distributed and compared between the intervention group and control group using the independent two-sample *t*-test. The results showed no statistical difference in the total score of SAIL and its three dimensions between the two groups (*p* > 0.05), indicating the baseline spiritual health level of the two groups were comparable (see Table 2 for details).

**Table 2:** Baseline comparison of spiritual health between the two groups

Dimension	Intervention group (*n* = 30)	Control group (*n* = 26)	*t*	*p*
	±	±		
Connect with yourself	44.40 ± 7.27	45.88 ± 7.75	− 0.739	0.463
Connect with the environment	25.40 ± 3.63	24.92 ± 4.79	0.422	0.674
Connect with transcendence	26.97 ± 6.58	24.46 ± 7.01	1.378	0.174
Overall spiritual health	96.76 ± 15.08	95.27 ± 16.40	0.356	0.723

### Comparison of spiritual health between the two groups before and after intervention

The paired sample *t*-test was used to compare the scores of SAIL and its three dimensions before and after the intervention in the intervention group and control group, respectively. In the intervention group, as shown in Table 3, the scores of SAIL and its three dimensions were significantly higher after the intervention than before the intervention (*p* < 0.001). In the control group, as shown in Table 4, there was no significant difference in the scores of SAIL and its three dimensions before and after the intervention (*p* > 0.05).

**Table 3:** Comparison of spiritual health before and after intervention in the intervention group (*n* = 30)

Dimension	Before intervention	After intervention	*t*	*p*
	±	±		
Connect with yourself	44.40 ± 7.27	48.43 ± 6.49	17.395	< 0.001
Connect with the environment	25.40 ± 3.63	28.33 ± 3.09	16.390	< 0.001
Connect with transcendence	26.97 ± 6.58	30.17 ± 6.40	17.588	< 0.001
Overall spiritual health	96.76 ± 15.08	106.93 ± 13.82	− 29.380	< 0.001

**Table 4:** Comparison of spiritual health before and after the intervention in the control group (*n* = 26)

Dimension	Before intervention	After intervention	*t*	*p*
	±	±		
Connect with yourself	45.88 ± 7.75	45.84 ± 7.68	− 0.189	0.852
Connect with the environment	24.92 ± 4.79	24.80 ± 4.74	− 0.440	0.664
connect with transcendence	24.46 ± 7.01	24.65 ± 6.74	− 0.243	0.810
Overall spiritual health	95.27 ± 16.40	95.31 ± 16.24	− 0.189	0.852

### Comparison of spiritual health changes after the intervention between the two groups

The independent two-sample *t*-test was performed to compare the score changes of the SAIL and its three dimensions after intervention between the intervention group and the control group. As shown in Table 5, the two groups showed statistically significant differences in the scores of SAIL and its three dimensions (*p* < 0.001).

**Table 5:** Comparison of spirit health changes after intervention between the two groups

Dimension	Intervention group (*n* = 30)	Control group (*n* = 26)	*t*	*p*
	±	±		
Connect with yourself	4.03 ± 1.27	− 0.38 ± 0.45	16.403	< 0.001
Connect with the environment	2.93 ± 0.98	− 0.16 ± 0.51	14.231	< 0.001
Connect with transcendence	3.20 ± 0.99	0.19 ± 0.69	12.908	< 0.001
Overall spiritual health	10.17 ± 1.89	0.04 ± 1.04	24.257	< 0.001

## Discussion

### Summary of the findings

In this quasi-experimental study, a total of 60 patients with liver cancer were assigned to either a “health education” WeChat group as the control group or a “bibliotherapy” WeChat group as the intervention group. Their spiritual health was assessed by using SAIL and compared before and after the intervention between the two groups. The results showed significant group differences in spiritual health, with higher scores of the SAIL and its three dimensions in the intervention group than in the control group. Our findings indicated that bibliotherapy based on traditional Chinese culture could significantly improve the spiritual health of patients with liver cancer, reflected in the three dimensions of connection with yourself, connection with the environment, and connection with transcendence.

Our study contributes to the literature by adding further support for the efficacy of bibliotherapy in improving the spiritual health of cancer patients in China, apart from the well-established benefits in improving mental health and quality of life among cancer patients [12]. In addition, the application of the WeChat platform to deliver health intervention among cancer patients has demonstrated its acceptability, feasibility, and efficacy. This finding was also consistent with a literature review illustrating that the use of WeChat/WhatsApp on cancer management could improve various physical and psychosocial health outcomes among oncological patients [21]. A major advantage of the bibliotherapy intervention in this study was the combination of traditional Chinese culture including Confucianism, Taoism, and Traditional Chinese Medicine; the mechanism of each in promoting the spiritual health of cancer patients was discussed below.

### The influence of Confucianism on spiritual health

Confucianism attaches great importance to benevolence, which represents the quality of being kind and helpful, with an emphasis on moral cultivation [22]. Benevolence serves as the core of Confucian culture and cultivates people’s conscience and morality, which will help realize the health and harmony of people’s bodies, minds, and spirits [23]. The bibliotherapy based on Confucianism can increase the participants’ knowledge and understanding of Confucian traditional culture, which can effectively improve their moral cultivation, and enhance their kindness, thus leading to improved spiritual health.

### The influence of Taoism on spiritual health

Taoism advocates that humans should live in harmony with the universe [24]. Taoists believe in spiritual immortality and the afterlife, where the spirit of the body joins the universe after death [25]. As a result, people should not be bound by their pessimistic emotions, but instead, maintain a calm and pleasant state of mind and return to the simple essence [24]. Taoism helps individuals to maintain a more peaceful attitude toward life [25]. In this study, bibliotherapy based on Taoism can help cancer patients absorb and accept Taoist culture, achieve tranquility of mind, learn to accept the adversity in life, and realize harmony of body and mind, which all contribute to improved spiritual health.

### The influence of traditional Chinese medicine on spiritual health

Traditional Chinese medicine focuses on the balance between body and emotions and believes that emotions such as joy, anger, sadness, and joy are closely related to health [26]. It is proposed that excessive emotional reactions will cause qi and blood disorders and lead to diseases [27]. Therefore, traditional medicine advocates a peaceful mind and less anxiety, with a focus on emotional management. In this study, bibliotherapy based on traditional Chinese medicine can help participants deepen their understanding of traditional medicine and form a calm attitude toward life, thus leading to improved spiritual health.

## Limitations

The study had several limitations. First, this is a quasi-experimental study, and the results may not be as robust as a randomized controlled study. Second, the sample was recruited through the convenience sampling method and the sample size is relatively small, which may affect the result generalization. Third, we only evaluated spiritual health as the intervention outcome and there may be other health benefits that were not assessed here.

## Conclusions

In conclusion, our study showed that bibliotherapy based on a combination of Confucianism, Taoism, and Traditional Chinese Medicine using the WeChat platform can greatly improve the spiritual health of patients with liver cancer, reflected in the three dimensions of connection with yourself, connection with the environment, and connection with transcendence. Our findings suggest that bibliotherapy is a cost-effective and feasible health intervention program that has the potential to be widely applied to cancer patients to improve their well-being. Our results provide important implications for the development and popularization of WeChat-based bibliotherapy in health promotion. Future studies may build on our findings to further explore other health benefits of bibliotherapy among other populations.

## Data Availability

All the data and materials underlying this article are available in the article.
